# Pollinator‐mediated interactions between cultivated papaya and co‐flowering plant species

**DOI:** 10.1002/ece3.4781

**Published:** 2018-12-11

**Authors:** Raúl Badillo‐Montaño, Armando Aguirre, Miguel A. Munguía‐Rosas

**Affiliations:** ^1^ Laboratorio de Ecología Terrestre Cinvestav Mérida México; ^2^ Red de Interacciones Multitróficas Instituto de Ecología, A.C. Xalapa México

**Keywords:** crop pollination, facilitation, pollination, pollinator‐mediated interactions, post‐pollination events

## Abstract

Many modern crop varieties rely on animal pollination to set fruit and seeds. Intensive crop plantations usually do not provide suitable habitats for pollinators so crop yield may depend on the surrounding vegetation to maintain pollination services. However, little is known about the effect of pollinator‐mediated interactions among co‐flowering plants on crop yield or the underlying mechanisms. Plant reproductive success is complex, involving several pre‐ and post‐pollination events; however, the current literature has mainly focused on pre‐pollination events in natural plant communities. We assessed pollinator sharing and the contribution to pollinator diet in a community of wild and cultivated plants that co‐flower with a focal papaya plantation. In addition, we assessed heterospecific pollen transfer to the stigmatic loads of papaya and its effect on fruit and seed production. We found that papaya shared at least one pollinator species with the majority of the co‐flowering plants. Despite this, heterospecific pollen transfer in cultivated papaya was low in open‐pollinated flowers. Hand‐pollination experiments suggest that heterospecific pollen transfer has no negative effect on fruit production or weight, but does reduce seed production. These results suggest that co‐flowering plants offer valuable floral resources to pollinators that are shared with cultivated papaya with little or no cost in terms of heterospecific pollen transfer. Although HP reduced seed production, a reduced number of seeds per se are not negative, given that from an agronomic perspective the number of seeds does not affect the monetary value of the papaya fruit.

## 1 INTRODUCTION

Approximately 87% of flowering plant species (Ollerton, Winfree, & Tarrant, 2011) and 74%–84% of crops depend to some extent on animal pollination to set fruit and seeds (Chacoff, Morales, Garibaldi, Ashworth, & Aizen, 2010; Klein et al., 2007). The value of the service of animal pollination to global agriculture was approximately €153 billion in 2005 (Gallai, Salles, Settele, & Vassière, 2009). Although the area of cultivated, pollinator‐dependent crops has been generally increasing (ca. 300% since the late 1950s; Aizen, Garibaldi, Cunningham, & Klein, 2009), its productivity per unit of area has decreased mainly because of the global decline in pollination services (Gallai et al., 2009; Kluser & Peduzzi, 2007). Thus, an increase in the cultivated area alone is unlikely to satisfy future demand for food by the growing human population (Godfray et al., 2010). This is particularly true for pollinator‐dependent crops because an increase in arable land usually entails the loss of forest cover and with this, the elimination of valuable floral resources and suitable habitat for pollinators (Aizen et al., 2009; Garibaldi et al., 2014; Winfree, Aguilar, Vázquez, LeBuhn, & Aizen, 2009). Because the expansion of agriculture occurs at the expense of native vegetation, the interface between crops and wild plant species has dramatically increased (Goldewijk, 2001; Klein, Cunningham, Bos, & Stefan‐Dewenter, 2008). Therefore, it is crucial to understand the impact of pollinator‐mediated interactions between cultivated and wild plants on their reproductive success (Klein et al., 2008; Stanley & Stout, 2014).

Intensively grown crops usually offer an unsuitable habitat and limited ephemeral floral resources to pollinators (Nel et al., 2017); however, pollination services in these agroecosystems are often influenced by the structure of the landscape in which the crop is embedded (Klein et al., 2008; Kremen et al., 2007; Power, 2010; Ricketts, 2004; Stanley & Stout, 2014). Contemporary landscape mosaics may offer floral resources and suitable habitats to pollinators mainly in forest remnants or secondary vegetation located in the vicinity of crops (Bailey et al., 2014; Carvalheiro, Seymour, Nicolson, & Veldtman, 2012; Power, 2010). It is frequently believed that wild plant species co‐flowering with crops and pollinated by generalist insects facilitate the pollination success of nearby crops (Bailey et al., 2014; Blanche, Ludwig, & Cunningham, 2006, Chacoff & Aizen, 2006, Carvalheiro, Seymour, Veldtam, & Nicolson, 2010, Ricketts, 2004, but see: Chacoff, Aizen, & Aschero, 2008, Mayfield, 2005, Winfree, Williams, Gaines, Ascher, & Kremen, 2008). In fact, comprehensive meta‐analyses suggest that proximity to forest remnants increases pollinator richness, visiting rate, and stability (Garibaldi et al., 2011; Ricketts et al., 2008). However, surprisingly, proximity to forest remnants is a poor predictor of crop yield (Garibaldi et al., 2011; Ricketts et al., 2008). Why do more pollinator visits not translate into greater yield in crops closer to forests? This question has yet to be answered.

Pollination is a complex phenomenon, and its final effect on plant reproductive success is mediated by several pre‐ (e.g. pollinator visitation rate) and post‐pollination (e.g. pollen load quality, pollen–stigma interactions) events (Willcox, Aizen, Cunningham, Mayfield, & Rader, 2017). For instance, frequent pollinator visits may produce poor fruit/seed set if the pollen load is dominated by heterospecific pollen (hereafter HP) (Wilcock & Neiland, 2002). Previous studies looking at pollinator‐mediated interactions between wild and cultivated plant species have emphasized pre‐pollination events with little emphasis on post‐pollination events (e.g. Chacoff & Aizen, 2006, Winfree et al., 2008, Bailey et al., 2014). Such studies have also mainly focused on forest proximity (reviewed by Garibaldi et al., 2011, Ricketts et al., 2008), but there has been little attention to other mechanisms that could mediate the effect of co‐flowering on crop yield. Studies conducted in natural communities suggest that plant species in co‐flowering communities make a variable contribution to the diet of shared pollinators (Bergamo et al., 2017; Carvalheiro et al., 2014). As a result, HP is frequently transferred and deposited on the stigmas of the interacting plants (Ashman & Arceo‐Gómez, 2013; Morales & Traveset, 2008; Tur, Saez, Traveset, & Aizen, 2016). Contrary to the general belief (i.e. that wild plants facilitate crop pollination), the outcome of pollinator‐mediated interactions on the reproductive success of co‐flowering species in natural communities ranges from negative (competition) to positive (facilitation) (Arceo‐Gómez et al., 2016; Muchhala & Thomson, 2012; Tur et al., 2016). If crops co‐flower and share pollinators with wild plant species, it is not inconceivable that there may be positive, negative, or even neutral effects, on at least one step of the pollination process. In fact, pollinator sharing and HP transfer may be higher in crops than in wild species because the former have not coevolved with the native plant–pollinator network (Ashman & Arceo‐Gómez, 2013; Morales & Traveset, 2008).

In this study, we looked at pollinator‐mediated interactions between papaya (*Carica papaya*) cultivated on an experimental plantation and the surrounding co‐flowering plant community on the Yucatan Peninsula. Wild papaya populations are dioecious and therefore, highly pollinator‐dependent (Fuentes & Santamaría, 2014). Although modern papaya varieties can also produce self‐compatible hermaphrodite flowers, previous studies suggest that pollen deposition by pollinators on these flowers significantly increases fruit set and weight (Badillo‐Montaño, Aguirre, Santamaría, Martínez‐Natarén, & Munguía‐Rosas, 2018; Garrett, 1995; Martins & Johnson, 2009). Fruit set in the absence of pollinators is also less attractive to customers owing to the reduced size and round shape of the fruit (Martins & Johnson, 2009; Moo‐Aldana et al., 2017). In the study area, cultivated papaya blooms year round and is visited by a wide variety of generalist insects (Moo‐Aldana et al., 2017) that also visit many other wild and cultivated plant species around papaya plantations (Badillo‐Montaño et al., 2018). Therefore, pollinator‐mediated interactions among cultivated papaya and co‐flowering plant species are very likely. Using observational and experimental approaches, we dissected the pollination process to see how co‐flowering affects both pre‐ and post‐pollination events. Specifically, we assessed pollinator sharing, pollen transfer, and the effects of HP transfer on fruit and seed production in cultivated papaya. Our specific goals in this study were to: (a) identify pollinators shared between cultivated papaya and the surrounding co‐flowering plant species, (b) assess the extent to which co‐flowering plants contribute to the diet of shared pollinators, (c) determine the degree of HP transfer to cultivated papaya, and (d) determine experimentally whether or not HP pollen load affects the quantity and quality of the yield in this crop species.

## 2 MATERIALS AND METHODS

### 2.1 Study system

The study area is located in the municipality of Muna on the Yucatan Peninsula, Mexico (20° 24′ 35.59″ N, 89° 45′ 30.57″ W; 38 m a.s.l.). The climate is sub‐humid, warm with summer rains; the mean annual temperature is 25.3°C (Campos‐Navarrete, Abdala‐Roberts, Munguía‐Rosas, & Parra‐Tabla, 2015). The study area is located in a landscape mosaic that encompasses fragments of original forest, patches of secondary vegetation, crop plantations, and cattle pastures. The dominant wild plant species in this area are as follows: *Bursera simaruba, Caesalpinia gaumeri,* *Cordia dodecandra*, *Lysiloma latisiliquum, Merremia dissecta, Piscidia piscipula, Pithecellobium albicans, Tabebuia rosea, *and *Thouinia paucidentata *(Campos‐Navarrete et al., 2015). The main crops in the area are as follows: corn (*Zea mays*), lemon (*Citrus limon*), orange (*Citrus sinensis*), mango (*Mangifera indica*), and moringa (*Moringa oleifera*) (Badillo‐Montaño et al., 2018). Cultivated papaya coexists with its wild relative (Fuentes & Santamaría, 2014), the latter occurring sparsely within the area sampled. The co‐occurrence of wild and cultivated papaya is relevant because these varieties have similar floral morphology and share several pollinator species (Moo‐Aldana et al., 2017). Most plants in the study area (including papaya) have entomophilous flowers and are visited mainly by bees (Badillo‐Montaño et al., 2018).

Papaya is originally from Mesoamerica but is currently cultivated in several tropical and subtropical regions worldwide (Fuentes & Santamaría, 2014). It is also one of the most economically valuable tropical fruits in the world, and Mexico is its second largest exporter (Badillo‐Montaño et al., 2018). Papaya is a perennial giant herb, cultivated varieties have a short lifespan of 2–4 years, and start producing flowers at the age of 5 months (Fuentes & Santamaría, 2014). Papaya blooms year round with a flowering peak in the Yucatan from March to June (Badillo‐Montaño et al., 2018). While wild populations of papaya are dioecious, in cultivated varieties, in addition to male and female plants, andromonoecious plants (male and hermaphroditic flowers produced by the same plant) are also present (Badillo‐Montaño et al., 2018). In the study area, andromonoecy is the most common sexual expression and male is the rarest. Papaya is mainly pollinated by generalist diurnal insects (bees and Lepidoptera) in the study area (Badillo‐Montaño et al., 2018; Moo‐Aldana et al., 2017). Some bee species also visit papaya on other continents (Australia and Africa) where this crop was introduced; however, some authors have suggested that the main pollinators are hawk moths (Garrett, 1995; Martins & Johnson, 2009). Although hawk months visit papaya flowers in the study area, these are infrequent visitors (Moo‐Aldana et al., 2017) and the proboscis of the species reported in the area (*Cautethia yucatana* and *Manduca *sp. Montero‐Muñoz, Pozo, & Cepeda‐González, 2013) is far longer (6.5 ± 1.2 cm [hereafter mean values ±1 *SE*]) than the corollas of female flowers (2.1 ± 0.4 cm). Male flowers produce nectar and pollen while hermaphroditic flowers only produce pollen as a reward; apparently, female flowers are pollinated by deceit (Garrett, 1995). Regardless of the variety, the hermaphroditic flowers of cultivated papaya exhibit herkogamy and two morphotypes are clearly differentiated in experimental plants: flowers with short (anther‐stigma distance <2.5 mm) and long (>5 mm) herkogamy. Floral longevity of cultivated papaya in the study area is 2–3 days (Moo‐Aldana et al., 2017). While papaya in the plantation under study may set fruit without pollinator assistance; these fruit are 26% smaller and have 60% fewer seeds than fruit from open‐pollinated flowers (Badillo‐Montaño et al., 2018).

### 2.2 Experimental plantation

In the summer of 2015, a number of papaya seedlings were obtained from a plant nursery belonging to the *Instituto Nacional de Investigaciones Forestales Agrícolas y Pecuarias* (INIFAP). About 648 papaya seedlings were selected (vigorous, apparently healthy plants) and planted in an experimental plot approximately 0.5 ha in area. The plants were placed in groups of three seedlings along nine rows (24 groups per row), between‐group distance was approximately 3 m. Three different varieties were planted in the plot (Maradol, Msxj, and BS‐2, 72 groups of three seedlings for each variety), eight groups of three plants per variety were planted in each of the nine rows (*n* = 648 plants in total, 72 groups, 216 plants per variety). From each group of seedlings, two plants were removed when at least one plant had reached 0.5 m in height, leaving the healthiest plant of the group (plants with no or low indications of pathogens and/or herbivore attack). When plants started producing flowers, sexual expression per plant was recorded. In total, 186 andromonoecious, three males and 27 females were recorded in the experimental plot. This unusually low ratio of plants with unisexual flowers has been previously reported in papaya, this is partially due to lethal genes associated with sex chromosomes, and also due to artificial selection (Ming, Yu, & Moore, 2007). All plants were watered as needed and received periodic manual weeding and pathogen control with fungicides. No insecticide was applied to avoid undesired effects on pollinators. As usual in intensive papaya plantations, no pollinator nest or refuge was seen during the study within the plantation. The Maradol variety is the most common variety cultivated in the Yucatan (Moo‐Aldana et al., 2017), and the Msxj and BS‐2 varieties were developed by INIFAP to withstand high temperatures (Mirafuentes & Santamaría, 2014; Santamaría, Mirafuentes, & Azpeitia, 2015). According to the literature, the three varieties do not differ in the variables relevant to our study (sexual expression, floral rewards, floral display, and plant size) (Badillo‐Montaño et al., 2018; Mirafuentes & Santamaría, 2014; Santamaría et al., 2015).

### 2.3 Shared pollinators

A circular area ca. 177 ha with the experimental plantation at its center was delimited to record flower visitors. Floral resources (wild and cultivated plants) were scattered within this area; however, vegetation closer than 3 m or within the plantation was cleared. Thus, cultivated papaya was the only source of floral resources within the plantation, no cultivated papaya was observed outside of the plantation in the sampled area. A portion of two forest patches also fell within the sampled area. The radius of this area (751 m) was slightly smaller than the mean foraging distance (815 ± 10 m) of bees, with body size similar to species found in the study area (Araujo, Costa, Chaud‐Netto, & Fowler, 2004; Zurbuchen et al., 2010). From April through June 2016, floral visitors were recorded for three consecutive days, twice a month. Flower visitors were surveyed from 0700 to 1300 hr, the observed peak of activity for flower‐visiting insects (Badillo‐Montaño et al., 2018). Floral visitors were observed while we walked along four strategically located (i.e. near floral resources) transects (Hernández‐Yañez, Lara‐Rodríguez, Díaz‐Castelazo, Dáttilo, & Rico‐Gray, 2013). When a flowering individual was detected in any of the transects, the observer stopped and recorded all flower visitors on each focal plant for a period of 10–15 min. Floral visitors that touched the reproductive organs of the flowers were considered effective pollinators (Saez, Morales, Ramos, & Aizen, 2014; Vázquez, Morris, & Jordano, 2005). Floral visitors not identified in the field were collected with entomological nets and preserved in 70% ethanol or photographed for later identification.

### 2.4 Pollen transfer

Pollen transfer was assessed by counting the number of pollen grains deposited on the stigmas of cultivated papaya flowers. To do so, during spring and summer 2016, 30 randomly selected papaya flowers per variety (*n* = 90) were tagged and allowed to open‐pollinate. No more than one flower per plant was tagged simultaneously, but some plants were chosen more than once during the experiment. Flower stigmas were collected after 48 hr and immediately fixed in ethanol 70% (Arceo‐Gómez et al., 2016). Once in the laboratory, the stigmas were rehydrated with water, decolorized with NaOH (5 N) at 37°C for 12 hr, and stained with aniline blue 0.3% for 18 hr (Alonso et al., 2013). Then, the stigmas were placed on microscope slides and observed with a fluorescence microscope (Leica DM1000, Germany) under a 515–560 nm excitation filter at magnifications of 10×, 20×, and 40× (Kearns & Inouye, 1993). Based on pollen morphology, conspecific pollen (hereafter CP) and HP in stigmatic loads were identified and counted. Although the pollen of papaya can be identified easily, HP was not identified to the species level because pollen morphology is not species‐specific in some plant groups or differences are not observable with the technique we used.

### 2.5 Fruit production and seeds

To assess the effect of HP transfer on fruit and seed production, a hand‐pollination experiment was conducted. Thirty‐three hermaphroditic floral buds per variety were tagged and bagged with a mosquito net on different plants (*n* = 99 flowers and plants). Immediately after anthesis, 33 randomly selected flowers were hand‐pollinated with CP (control group). From the remaining 66 flowers, a random subgroup of 33 flowers (treatment 1) was hand‐pollinated with a mix of CP from the plantation and pollen from *M. dissecta* and *M. oleifera*. To do so, we first harvested the pollen of as many anthers as possible per species, and then we took approximately the same amount of pollen in volume of each species and mixed it to obtain a homogenous mixture. Therefore, the proportion of pollen per species in the mixture used for hand pollinations was approximately 1:1:1. A second subgroup of 33 flowers (treatment 2) was hand‐pollinated with a mix of CP from the plantation, pollen from wild papaya, and pollen from *M. dissecta* (1:1:1), following the procedure described for treatment 1. Pollen from wild papaya was used in treatment 2 because it is known that cultivated varieties share some pollinator species with wild papaya when they co‐occur (Moo‐Aldana et al., 2017). *M. dissecta* and *M. oleifera* were selected because they co‐flower with cultivated papaya, share some pollinator species (3) with cultivated papaya, and produce abundant and accessible pollen. The same number of flowers (11) per variety and per treatment was selected. Before pollen was placed on the stigma, flowers were carefully emasculated. In all cases, pollen was placed on the stigma until it was saturated. After hand‐pollinating the flowers, all flowers were bagged with a mosquito net. Fruit set was recorded weekly and, once ripe, fruit were weighed and seeds counted.

### 2.6 Data analyses

Sampling completeness of flower–visitor interactions was assessed by comparing the number of observed and expected interactions based on the Chao 2 estimator and pooling the data of all plant species (Chacoff et al., 2012). To visually analyze the structure of the co‐flowering plant–pollinator network, we built a quantitative plant–pollinator network using the bipartite package for R (R Core Team, 2017). Then, we ran a hierarchical cluster analysis based on among‐plant dissimilarity (Bray‐Curtis) in terms of the pollinator species using the average agglomerative method (Everitt & Hothorn, 2011; Goslee & Urban, 2007). Cluster uncertainty was assessed with bootstrap resampling methods (1,000 replicates) implemented in the pvclust package of R 3.3.3. (Suzuki & Shimodaira, 2006). While the cluster analysis allowed us to identify similarity between plant species in terms of pollinator identity and frequency, for a given pair of plant species, the influence (i.e. pollen transfer) of one species (acting plant) on the other (target plant) may be asymmetrical (Bergamo et al., 2017). To address potentially asymmetrical pollinator‐mediated interactions among pairs of co‐flowering plant species, we calculated Müller's index (Müller, Adriaanse, Belshaw, & Godfray, 1999) between cultivated papaya and all co‐flowering species. Müller's index (*d*_ij_) was defined as:

$$d_{\mathrm{ij}}\;=\;\sum_{k}\;\left[\frac{\alpha_{\mathrm{ik}}}{\sum_{l}\alpha_{\mathrm{il}}}\;\times \;\frac{\alpha_{\mathrm{jk}}}{\sum_{m}\alpha_{\mathrm{mk}}}\right]$$

where *α_ik_* represents the number of interactions of pollinator *k* to the target plant species *i* (*l = *total number of pollinators to the target plant species), and *α_jk_* represents the number of interactions of pollinator *k* with the acting plant species *j *(*m* = total number of plants with which pollinator *k* interacts). To estimate the indirect influence of the pollinators of cultivated papaya on the co‐flowering community and vice versa, the index was calculated first with cultivated papaya as the acting plant and then as the target plant. In the context of pollinator‐mediated interactions, Müller's index is also a proxy for how much each of the acting plants contributes to the diet of all pollinators shared with each target plant (Bergamo et al., 2017; Carvalheiro et al., 2014; Nel et al., 2017). Müller's index goes from zero (no pollinator sharing, small contribution to the diet of shared pollinators) to one (all pollinator are shared, large contribution to the diet of shared pollinators) (Bergamo et al., 2017; Carvalheiro et al., 2014; Nel et al., 2017). Müller's index was calculated with the PAC function implemented in the bipartite packaged for R.

To assess the indirect effect of co‐flowering plant species on pollen transfer to cultivated papaya, a mixed‐effects generalized linear model with a negative binomial error distribution and the logarithmic link function was fitted. In this model, the number of CP in stigmatic loads of cultivated papaya was the response variable and the number of HP was the explanatory variable (Tur et al., 2016). Additionally, the flower morph (a three‐level factor: hermaphroditic flowers with short herkogamy, hermaphroditic flowers with long herkogamy and female flowers) and its interaction with the number of HP were included as explanatory variables in the model. The effect of hand‐pollination treatments on fruit set (a dichotomous variable) was assessed with a generalized linear mixed‐effects model with binomial error distribution and logit link function. To assess the effect of the same treatments on fruit weight and seed number, a linear mixed‐effects model (Gaussian error) and a generalized mixed‐effects model (Poisson error, log link function) were fitted, respectively. The variety of cultivated papaya was included as a random factor to account for any among‐variety variation in all models.

## 3 RESULTS

### 3.1 Shared pollinators

**Figure 1:**
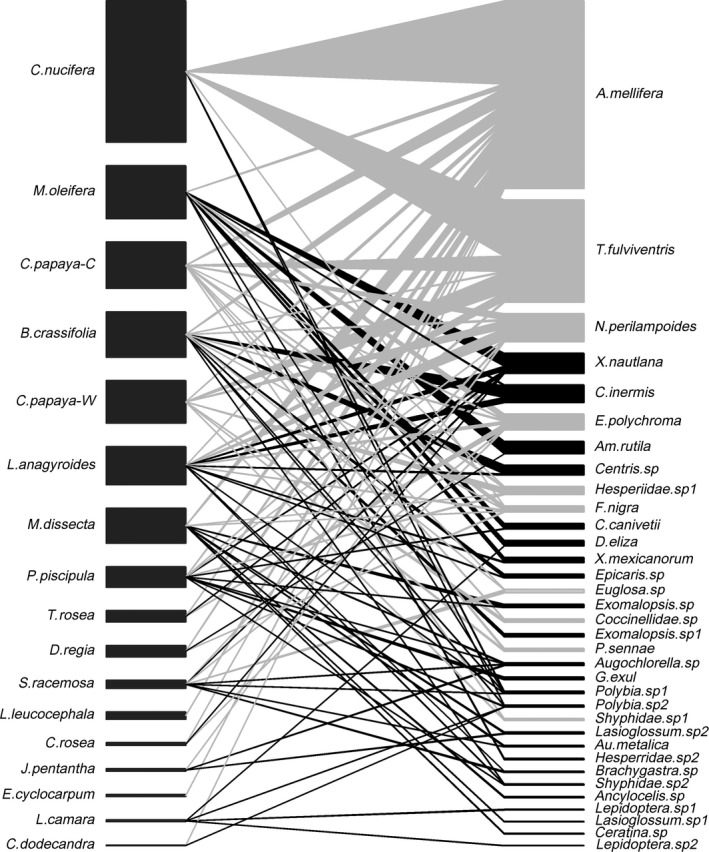
Plant–pollinator network showing the interactions between co‐flowering plant species in a landscape mosaic in Muna, Yucatan, Mexico. Pollinators are on the right and plants on the left. Bar heights indicate relative pollinator visits (plants) and relative visiting rate (pollinators). Nodes of pollinators that visited cultivated papaya (C papaya‐C) and links to plants sharing at least one pollinator species with cultivated papaya are in gray

During the study, 18 plant species co‐flowered with cultivated papaya, and all of them shared at least one pollinator species with cultivated papaya (Figure 1), except for two *Citrus* species (lemon and orange) that were not visited. Thirty‐four pollinator species and 5, 201 flower visits were recorded. The most frequent pollinators were *Apis mellifera *(39.4%), and the native bee species *Trigona fulviventris *(21.5%) and *Nannotrigona perilampoides* (5.9%). The remaining 33.2% of visits were by social, eusocial, and solitary bee species (21 species), some species of Lepidoptera (5), Diptera (2), and Coleoptera (1), as well as hummingbirds (2). The most common pollinator species (*A. mellifera*, *N. perilampoides*, and *T. fulviventris*) were also the most frequently shared pollinators among co‐flowering plant species (Figure 1). Sampling completeness for flower–visitor interactions was 70%.

**Figure 2:**
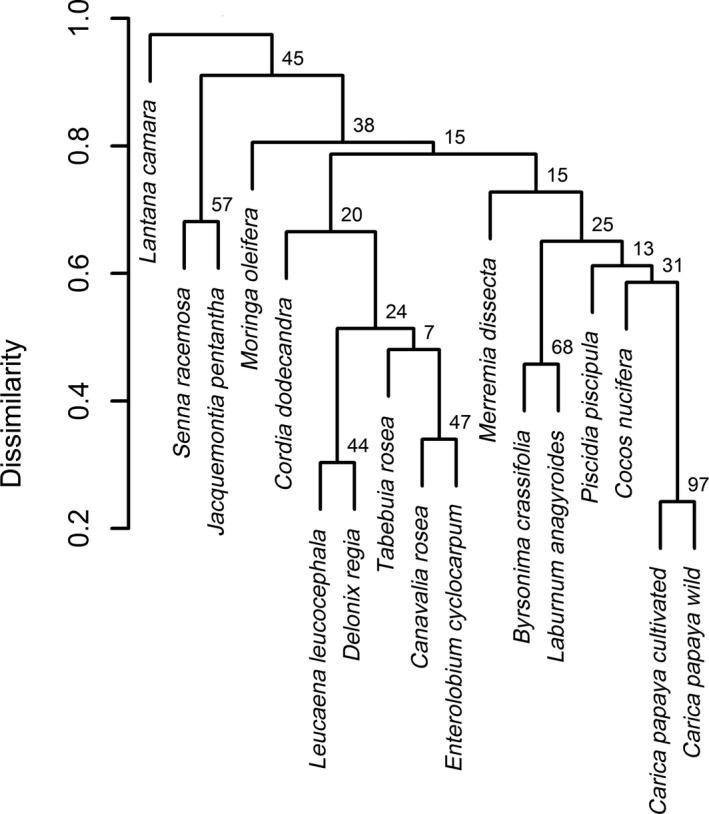
Hierarchical cluster of dissimilarity for pollinator assemblages in a community of co‐flowering plant species in a landscape mosaic on the Yucatan Peninsula. Values at the nodes are the times (in percentage) that a focal cluster appeared in 1,000 bootstrap iterations

**Figure 3:**
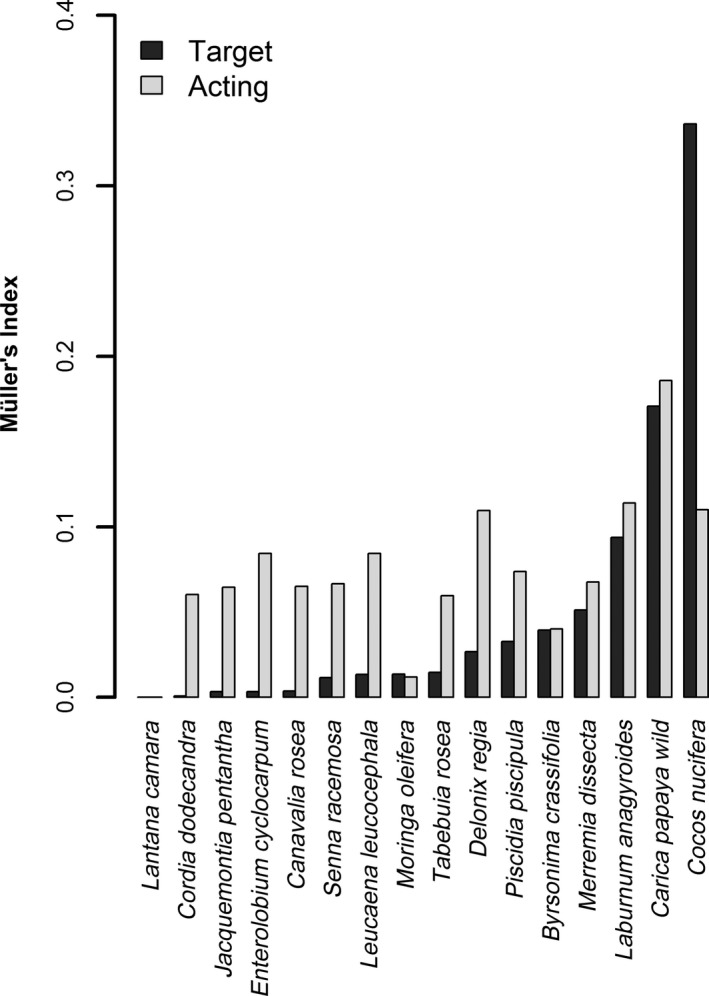
Müller's index for a community of co‐flowering plant species in a landscape mosaic on the Yucatan Peninsula. Black bars represent the index when cultivated papaya is the target species and white bars when cultivated papaya is the acting species

Wild papaya was the co‐flowering plant most similar to cultivated papaya in terms of pollinators (seven shared pollinator species), followed by *Byrsonima crassifolia* (six shared species), *Laburum anagyroides* (five shared species), *P. piscipula* (four shared species), *C. nucifera* (three shared species), and *M. dissecta *(three shared species), all of which were grouped in the same cluster. *M. oleifera* also shared three pollinator species but was grouped in a different cluster owing to differences in visiting frequency (Figure 2). A relatively strong contribution to the diet of shared pollinators and a symmetrical influence was found between cultivated and wild papaya (Müller's index = 0.17 [cultivated papaya as target species] and 0.18 [cultivated papaya as acting species]) and between cultivated papaya and *L. anagyroides* (Müller's index = 0.10 [target] and 0.11 [acting]). Strong, asymmetrical interactions occurring in opposite directions, were detected between cultivated papaya and *C. nucifera* (Müller's index = 0.33 [target] and 0.11 [acting]) and between cultivated papaya and *D. regia* (Müller's index = 0.02 [target] and 0.11 [acting]). That is, the influence of *C. nucifera* on cultivated papaya, and that of cultivated papaya on *D. regia,* is stronger than in the opposite direction. With the exceptions of *C. nucifera* and *M. oleifera*, the pollinator‐mediated influence of cultivated papaya was slightly stronger as an acting (Muller's index = 0.07 ± 0.01) than as a target (0.05 ± 0.02) species (Figure 3).

### 3.2 Pollen Transfer

**Figure 4:**
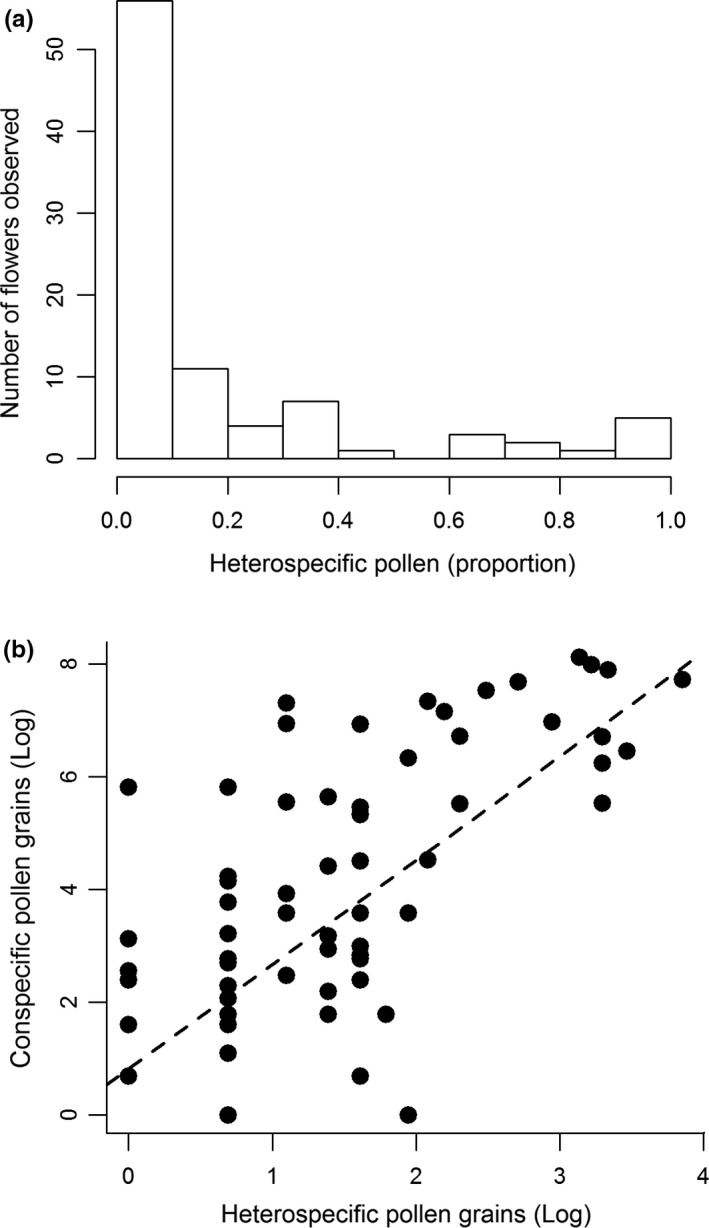
(A) Frequency distribution of heterospecific pollen grains (proportion) on the stigmas of flowers of cultivated papaya. (B) Relationship between the number of heterospecific and conspecific pollen grains found on the stigmas of the flowers of papaya cultivated in an experimental plot on the Yucatan Peninsula. The slope of the regression line was statistically different from zero. The values of the axes are shown on a log scale. Data shown in (A) and (B) are from the same sample (*n* = 90 flowers)

The mean number of CP was 331 ± 74.04 grains per stigma, while for HP it was 5.5 ± 1.89 grains per stigma. Proportionally, CP averaged 83 ± 4% and HP 17 ± 4%. However, in both cases (CP and HP), values ranges from 0% to 100%. About 79% of examined stigmas had some HP. And, only in 21% of these stigmas did HP represent ≥30% of the total pollen load (Figure 4a). The number of CP on the stigmas of cultivated papaya was significantly explained by the number of HP, and the relationship between these variables was positive (*β* = 0.16 ± 0.08, $\chi_{1}^{2}$ = 16.81, *p* < 0.001, explained deviance = 55%; Figure 4b). CP on the stigmas of female (163.60 ± 61.42) and hermaphrodite flowers with short (507 ± 167.51) and long (322 ± 128.84) herkogamy were not statistically different ($\chi_{2}^{2}$ = 3.07, *p = *0.21). The flower type × HP interaction was not statistically significant ($\chi_{2}^{2}$ = 1.88, *p = *0.38).

### 3.3 Fruit production and seeds

Flowers that were hand‐pollinated with a mix of CP and HP (treatments 1 and 2) and CP alone (control) did not statistically differ in fruit set ($\chi_{2}^{2}$ = 0.99, *p = *0.95) or fruit weight (*F*_2,50 = _1.12, *p = *0.33). However, a difference among treatments was found in seed number per fruit ($\chi_{2}^{2}$ = 42.01, *p < *0.001). Flowers pollinated with a mix of HP and CP produced significantly fewer seeds than flowers pollinated only with CP (Table 1).

**Table 1:** Mean values (±1 SE) for fruit set, fruit weight and number of seeds per fruit of cultivated papaya under three hand‐pollination treatments (Conspecific, Heterospecific‐1 and Heterospecific‐2). Treatment Heterospecific‐1 consisted of a mix of conspecific pollen and pollen from *Merremia dissecta* and *Moringa oleifera* (1:1:1). Treatment Heterospecific‐2 consisted of a mix of conspecific pollen from the plantation, pollen from wild papaya and pollen from *M. dissecta* (1:1:1). Different superscript letters indicate statistically significant differences between treatments

Response	Treatment		
	Conspecific	Heterospecific−1	Heterospecific−2
Fruit set (%)	54 ± 8.1^a^	57 ± 8.1^a^	54 ± 8.0^a^
Fruit weight (kg)	1.45 ± 0.2^a^	1.35 ± 0.1^a^	1.23 ± 0.1^a^
Seeds (number)	454 ± 66^a^	383 ± 60^b^	322 ± 39^b^

## 4 DISCUSSION

In this study, we have shown that cultivated papaya shares at least one pollinator species with the majority of co‐flowering plants in the vicinity of an intensive experimental plantation. Despite this extensive pollinator sharing, observed HP transfer in cultivated papaya tends to be low. HP loads had no negative effect on fruit production or weight; even though experimental HP loads were greater than the loads on open‐pollinated flowers. We suggest that the effect of co‐flowering plants on crop yield of papaya is positive because these plants contribute to the diet of shared pollinators with little cost in terms of HP transfer.

Cultivated papaya shared at least one pollinator species with 88% of the co‐flowering plant species in the study area. Therefore, we think that cultivated papaya has been successfully integrated into the plant–pollinator network of co‐flowering plants, and this may have occurred because this crop has a generalist pollination system (Moo‐Aldana et al., 2017). For instance, cultivated papaya was visited by *A. mellifiera* and *T. fulviventris*, which were the most common pollinator species in the whole network, and thus, the visit of these two bee species alone would result in its successful incorporation into the network. Although nonnative plants have not coevolved with native flora and pollinators, previous studies suggest that a large floral display as well as a generalist pollination system may facilitate their integration into the plant‐pollination network (Jakobsson, Padron, & Traveset, 2008; Memmot & Waser, 2002). In the specific case of our study system, we also think that the presence of wild papaya in the study area may have played a role in the incorporation of cultivated papaya because the local pollinator fauna were already familiar with floral resources offered by cultivated papaya owing to the similarity in the floral traits that attract pollinators (Moo‐Aldana et al., 2017).

The results of the hierarchical clustering and Müller's index coincided in that cultivated papaya has a relatively strong pollinator‐mediated interaction with wild papaya, *C. nucifera* and *L. anagyroides* (Figures 2 and 3). This was also probably due to floral similarity between wild and cultivated papaya. For *C. nucifera* and *L. anagyroides*, this probably occurred because these plant species have massive floral displays (Meléndez‐Ramírez et al., 2004; Stawiarz & Wróblewska, 2013) that attract a widely diverse and abundant assemblage of generalist insect visitors, many shared with cultivated papaya. Müller's index also indicates a slightly stronger influence of papaya as an acting than as a target species, a finding that needs further attention. If co‐flowering plants actually receive pollen from cultivated papaya, this may lead to CP loss (Morales & Traveset, 2008) as well as the transfer of papaya pollen to wild plant species with potentially negative effects on their reproductive success (Stanley & Stout, 2014).

Also based on Müller's index, we expected a large quantity of HP on the stigmas of cultivated papaya, especially from *C. nucifera*, *L. anagyroides,* and *M. dissecta* (see black bars in Figure 3). However, even considering the pollen of all these species and the pollen of other unidentified species, the majority of stigmas examined (60%) had pollen loads where HP represented only 10% or less (see Figure 4a). From the perspective of cultivated papaya, this is advantageous because co‐flowering plants are contributing to the diet of shared pollinators but with little cost in terms of HP transfer. The papaya plantation could not maintain this pollination assemblage on its own because it does not provide a suitable habitat for pollinators. Some mechanisms have evolved to reduce HP transfer among co‐flowering plants in natural communities such as character displacement and pollinator partitioning (Muchhala & Potts, 2007; Stone, Willmer, & Rowe, 1998). Some of these may apply to plants co‐flowering with cultivated papaya because these plant species have coexisted with the wild relative of cultivated papaya in the study area and therefore, have coevolved with the network of native co‐flowering plants. The high density of floral resources typically found in monocultures may also explain the low levels of HP transfer (Ekroos et al., 2015; de Waal, Anderson, & Ellis, 2015). That is, to reduce foraging cost, pollinators may move preferentially within the plantation. The transfer of CP not assisted by pollinators (i.e. autonomous self‐pollination) is likely to be negligible in the plantation because CP load on stigmas was similar between female and hermaphrodite flowers.

Positive covariation between CP and HP on the stigmas of plants, as we observed in cultivated papaya, has been interpreted as evidence of facilitative interactions between co‐flowering plants (Tur et al., 2016). The rationale behind this is that when a community of co‐flowering plants contributes to the diet of shared pollinators, it increases pollen transfer in general (Tur et al., 2016). However, we cannot consider this to be evidence of facilitation if HP reduces plant reproductive success; an aspect rarely evaluated in co‐flowering plant communities (e.g. Carvalheiro et al., 2014, Tur et al., 2016, Bergamo et al., 2017). Using a proportion of HP of about 66%, we did not detect any effect of HP on fruit set or weight in cultivated papaya. In our sample, 87% of stigmas had a proportion of HP ≤66%. Therefore, the degree of HP transfer typically seen in open‐pollinated flowers has no negative effect on fruit production or quality. In one sense, the results of our experiment can be seen as an exacerbated effect of HP loads. Although seed production does not affect the economic value of papaya, the observed negative effect of high proportions of HP on seed production indicates that HP affects a post‐pollination process related to ovule fertilization and/or seed development (Aizen & Harder, 2007; Wilcock & Neiland, 2002). This may be relevant for other crops where seed yield is of primary interest to farmers, such as sunflower (Nderitu, Nyamasyo, Kasina, & Oroje, 2008) and almond (Dag, Zipori, & Pleser, 2006).

Although we have assessed the consequences of co‐flowering on the different pre‐ and post‐pollination events in cultivated papaya, we recognize that more effort is needed to bridge these pollination events. The identification of HP to the species level for all the co‐flowering species may help to link pollinator sharing and HP transfer; however, this may require a comprehensive reference collection of pollen and/or the use of more sophisticated techniques of pollen identification (e.g. scanning electron microscopy, and spectroscopy). Experiments emulating the actual quantity and quality (i.e. species of origin) of HP on stigmas would be the optimal approach for demonstrating a link between HP transfer and reproductive success; however, this may be challenging in systems where the size and composition of stigmatic loads in open‐pollinated flowers exhibit marked variation.

To summarize, cultivated papaya co‐flowered and shared pollinators with the majority of co‐flowering plants in the study area, thus we suggest that co‐flowering plants provide pollinators with supplementary floral resources. Despite extensive pollinator sharing, HP transfer was low. The presence of HP in stigmatic loads does not lead to reduced fruit set or fruit weight. Therefore, we conclude that the presence of co‐flowering plant species around a focal papaya plantation has a positive effect on the pollination success of the plantation via the provision of floral resources to shared pollinators. Although we detected a negative effect of HP on seed production, the number of seeds produced does not affect the sellable crop yield. Because we used a bigger proportion of HP in our experiments than observed in open‐pollinated flowers, we cannot rule out the possibility that HP in open‐pollinated flowers may have a lower or no effect on seed production.

## CONFLICT OF INTEREST

The authors have no conflict of interest to disclose.

## AUTHOR CONTRIBUTION

MAM‐R, RB‐M, and AA conceived the research ideas; RB‐M and MAM‐R collected data; RB‐M and MAM‐R analyzed data; RB‐M, MAM‐R, and AA wrote the manuscript and approved final content.

## DATA ACCESSIBILITY

Data used in this study is available in Dryad https://doi.org/10.5061/dryad.4rs855v.
